# Supplementary material for: Ecology-guided prediction of cross-feeding interactions in the human gut microbiome
Source: Nat Commun. 2021 Feb 26;12:1335. doi: 10.1038/s41467-021-21586-6 (PMC7910475; doi:10.1038/s41467-021-21586-6)
Supplement: Supplementary file 1 — Supplementary Information [file 41467_2021_21586_MOESM1_ESM.pdf]

Supplementary Figures
Ecology-guided prediction of cross-feeding interactions in the human gut microbiome

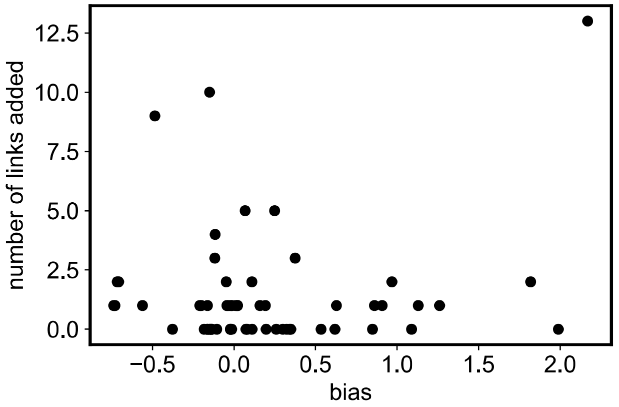

Supplementary Figure 1: No correlation between the prior metabolite systematic bias and the number links added related to the metabolite. Each point represents a metabolite. Results are shown for one run of GutCP. The Pearson correlation coefficient between the two quantities is 0.15, and the P value (two-sided t-test) is 0.24, which is not significant.

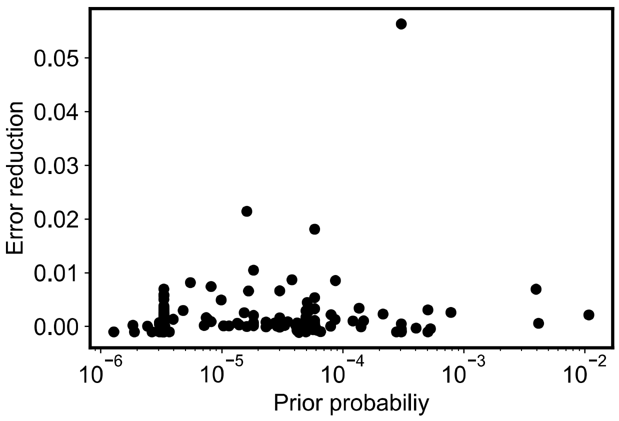

Supplementary Figure 2: No correlation between the prior metabolite probability and the error reduction induced by the added links related to the metabolite. Each point represents a metabolite. Results are shown for one run of GutCP. The Pearson correlation coefficient between the two quantities is 0.03, and the P value (two-sided t-test) is 0.74, which is not significant.

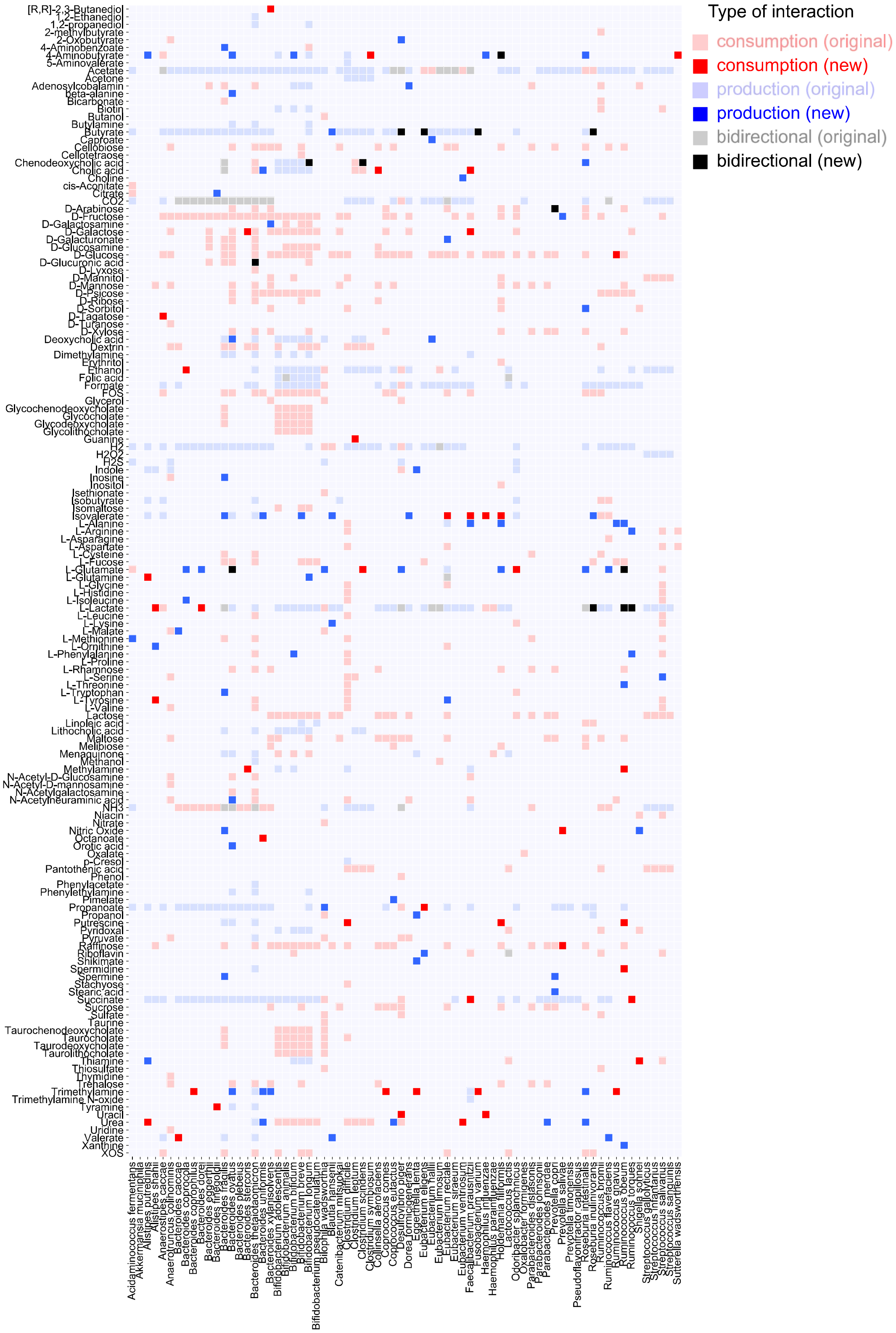

Supplementary Figure 3: A full cross-feeding network, similar to figure 3a, for all metabolites and microbes.

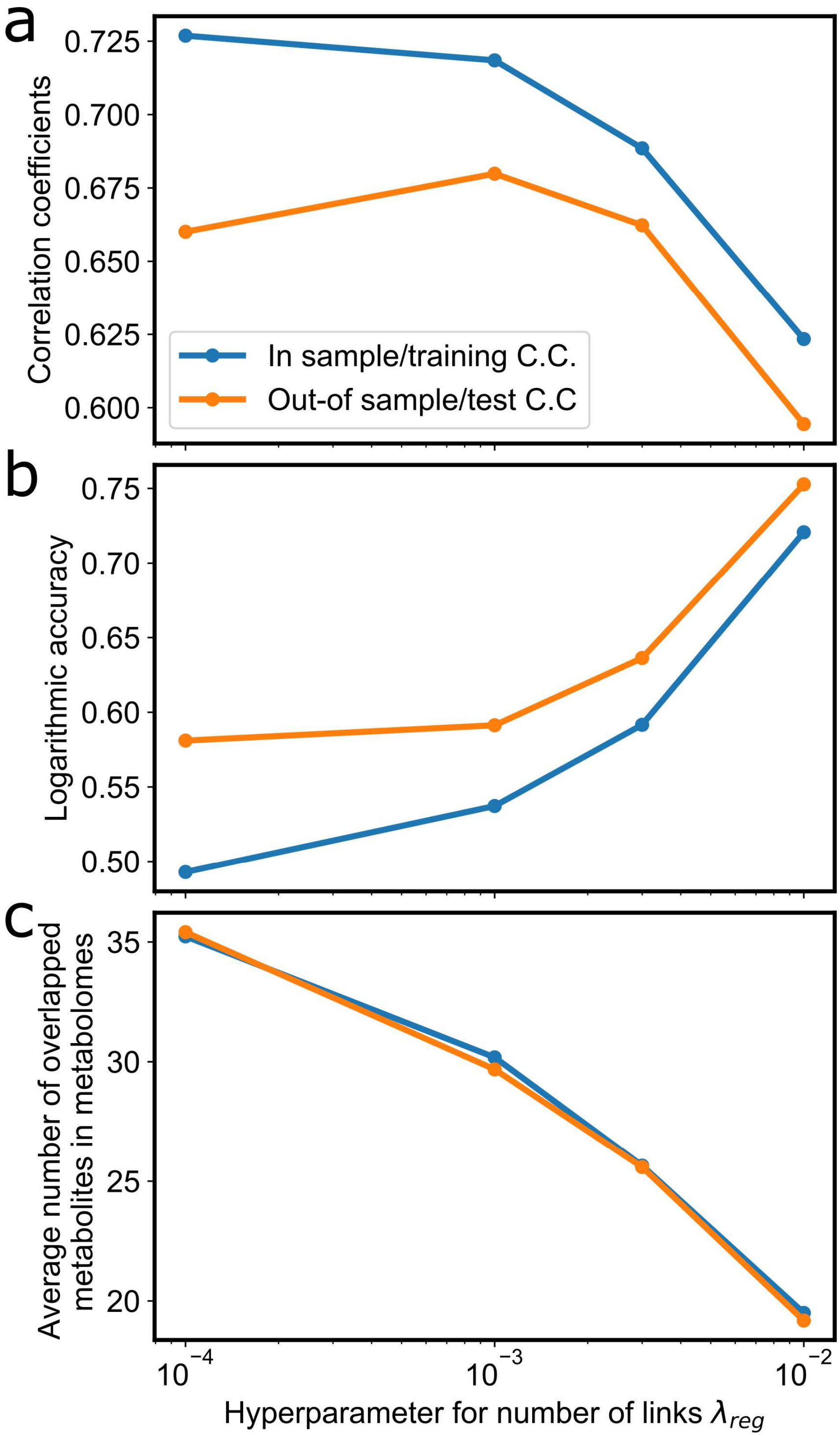

Supplementary Figure 4: The effect of the hyper-parameter for the number of links $\lambda_{\mathbf{reg}}$ on model performance. Results are shown for one run of GutCP. The other hyper-parameter for rewarding the number of overlapped metabolites $\lambda_{\mathrm{reward}}$ is fixed as 10^{-3} (see Methods).

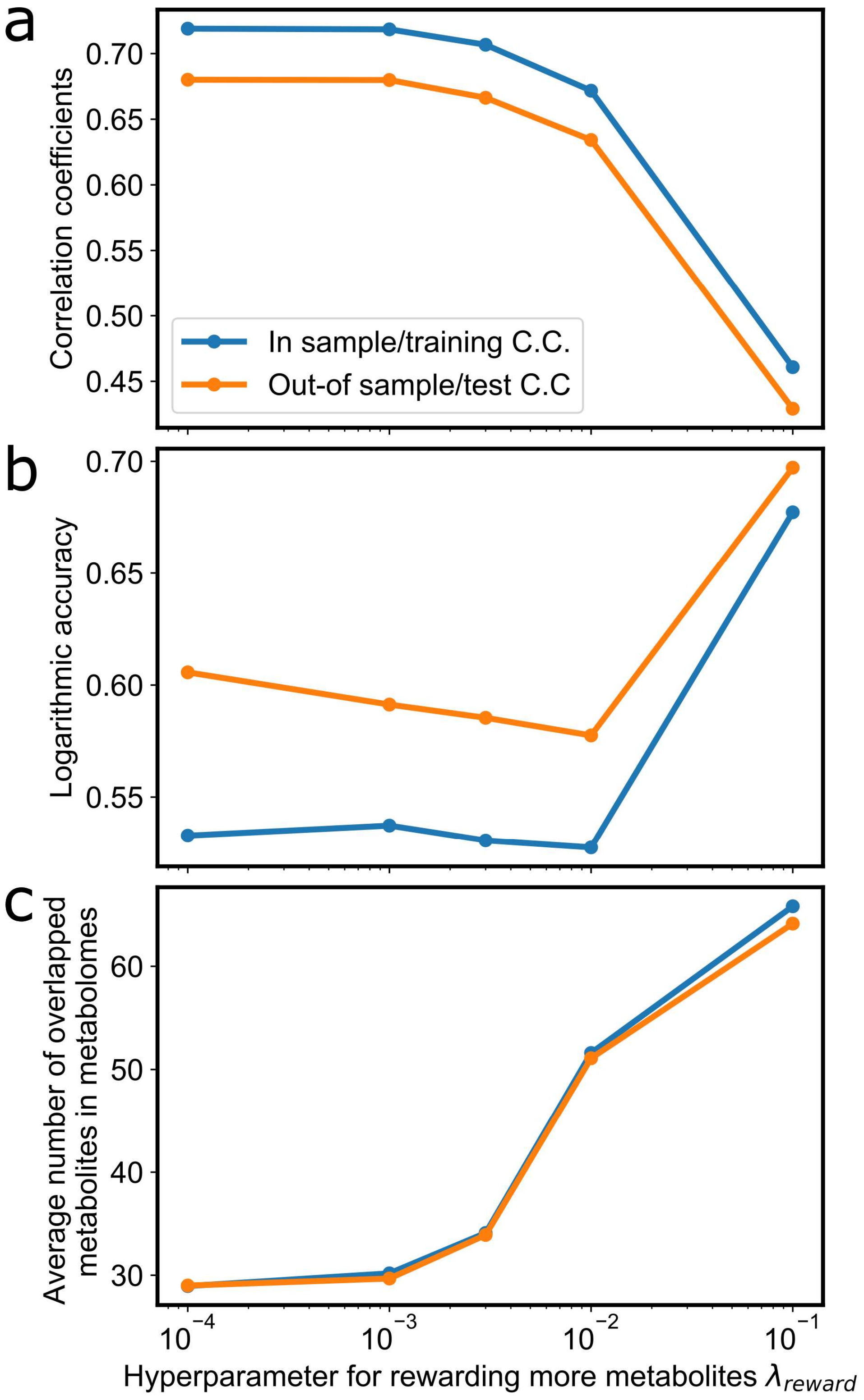

Supplementary Figure 5: The effect of the hyper-parameter for rewarding the number of overlapped metabolites $\lambda_{\mathbf{reward}}$ on model performance. Results are shown for one run of GutCP. The other hyper-parameter for the number of links λ_{reg} is fixed as 10^{-3} (see Methods).

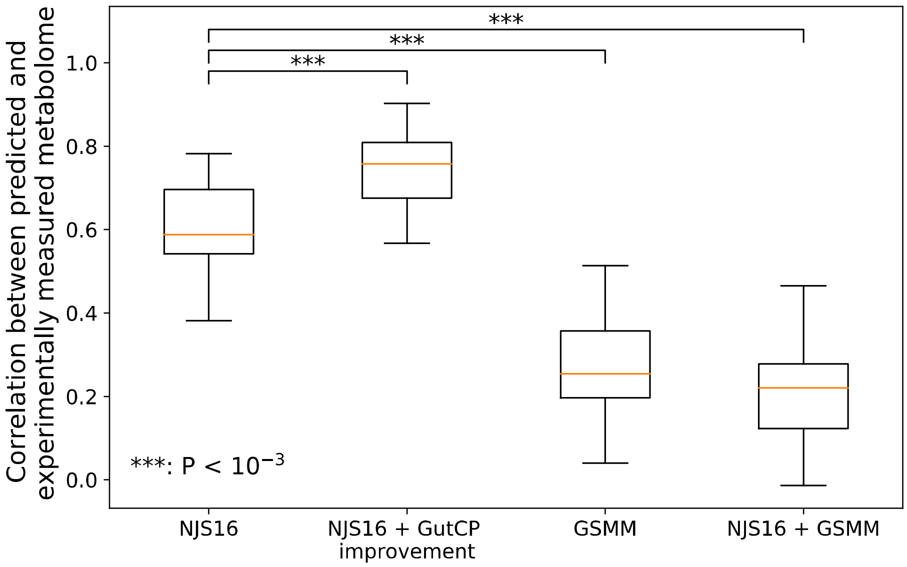

Supplementary Figure 6: The usage of metabolite-microbe consumption/production interactions from GSMM (Genome-Scale Metabolic Models) generates worse performance compared with that from manually curated NJS16 database (n = 41 independent samples). The p-values are generated by the two-sided Kruskal–Wallis test. Three p-values from left to right are 1.92×10^{-7}, 1.66×10^{-14} and 9.30×10^{-15}. In all boxplots, the middle line is the median, the lower and upper hinges correspond to the first and third quartiles, the upper whisker ranges from the hinge to the value 1.5 × IQR (where IQR is the interquartile range) above the hinge and the lower whisker extends from the hinge to the value 1.5 × IQR below the hinge, while all data points failing beyond the range of whiskers are plotted individually.

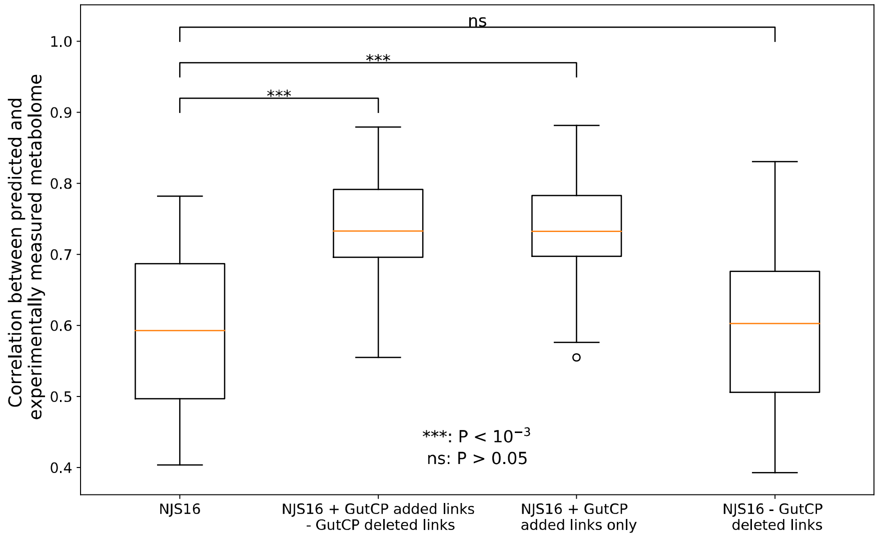

Supplementary Figure 7: The contribution of deleted links to the improvement of predictions is significantly smaller than that of added links. GutCP is modified to have the capability of deleting random links. Added or deleted links are selected over the process of simulation. When a simulation is done, a list of added links and a list of deleted links are generated separately. When only deleted links are removed from the NJS16 links (n = 41 independent samples), the performance improvement is not significantly according to the two-sided Kruskal–Wallis test. Three p-values from left to right are 1.49×10^{-7}, 2.01×10^{-7} and 0.71. In all boxplots, the middle line is the median, the lower and upper hinges correspond to the first and third quartiles, the upper whisker ranges from the hinge to the value $1.5 \times$ IQR (where IQR is the interquartile range) above the hinge and the lower whisker extends from the hinge to the value $1.5 \times$ IQR below the hinge, while all data points failing beyond the range of whiskers are plotted individually.

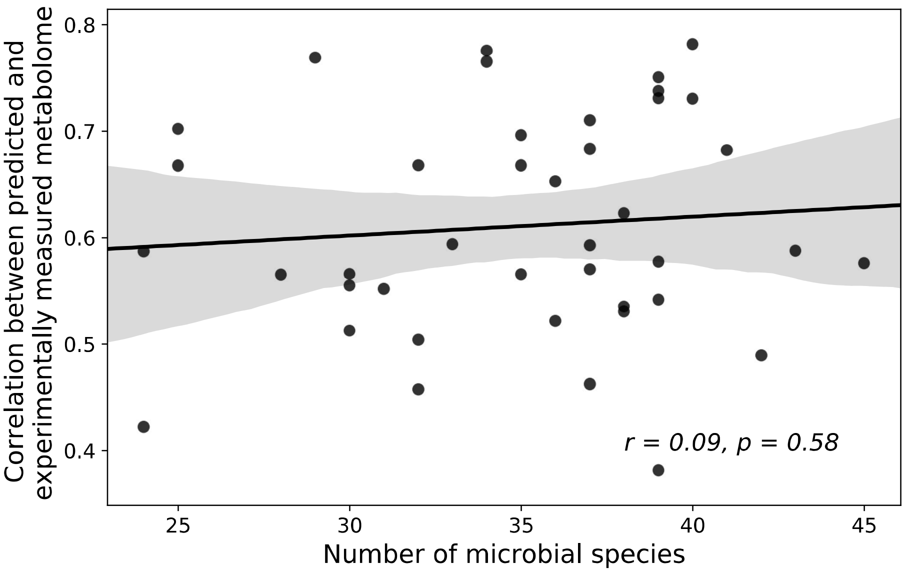

Supplementary Figure 8: The prediction accuracy doesn't correlate with the number of overlapped microbial species. The p-value for the Pearson correlation coefficient is generated by the two-sided t-test.

Supplementary Data Legends

Supplementary Data 1: Table containing all 293 consensus-based cross-feeding interactions predicted by GutCP.

Supplementary Data 2: Table containing all extracellular reactions extracted from genome-scale metabolic models (GSMMs) used to validate GutCP.
