# Supplementary material for: Ecology-guided prediction of cross-feeding interactions in the human gut microbiome
Source: Nat Commun. 2021 Feb 26;12:1335. doi: 10.1038/s41467-021-21586-6 (PMC7910475; doi:10.1038/s41467-021-21586-6)
Supplement: Supplementary file 6 — Description of Additional Supplementary Files [file 41467_2021_21586_MOESM6_ESM.pdf]

Title: Supplementary Data 1

Description: Table containing all 293 consensus-based cross-feeding interactions predicted by GutCP.

Title: Supplementary Data 2

Description: Table containing all extracellular reactions extracted from genome-scale metabolic models (GSMMs) used to validate GutCP.
